# The brain cytokine orchestra in multiple sclerosis: from neuroinflammation to synaptopathology

**DOI:** 10.1186/s13041-024-01077-7

**Published:** 2024-01-23

**Authors:** Roberta Amoriello, Christian Memo, Laura Ballerini, Clara Ballerini

**Affiliations:** 1 International School for Advanced Studies (SISSA/ISAS), 34136 Trieste, Italy; 2 Dipartimento di Medicina Sperimentale e Clinica, University of Florence, 50139 Florence, Italy

**Keywords:** Cytokines, Chemokines, Neuroinflammation, Multiple Sclerosis, Experimental autoimmune encephalomyelitis, Neuromodulation, Synaptopathy

## Abstract

The central nervous system (CNS) is finely protected by the blood–brain barrier (BBB). Immune soluble factors such as cytokines (CKs) are normally produced in the CNS, contributing to physiological immunosurveillance and homeostatic synaptic scaling. CKs are peptide, pleiotropic molecules involved in a broad range of cellular functions, with a pivotal role in resolving the inflammation and promoting tissue healing. However, pro-inflammatory CKs can exert a detrimental effect in pathological conditions, spreading the damage. In the inflamed CNS, CKs recruit immune cells, stimulate the local production of other inflammatory mediators, and promote synaptic dysfunction. Our understanding of neuroinflammation in humans owes much to the study of multiple sclerosis (MS), the most common autoimmune and demyelinating disease, in which autoreactive T cells migrate from the periphery to the CNS after the encounter with a still unknown antigen. CNS-infiltrating T cells produce pro-inflammatory CKs that aggravate local demyelination and neurodegeneration. This review aims to recapitulate the state of the art about CKs role in the healthy and inflamed CNS, with focus on recent advances bridging the study of adaptive immune system and neurophysiology.

## Introduction

Cytokines (CKs) are peptide molecules that orchestrate a complex and finely regulated network with a critical role in immunomodulation, exerting anti- or pro-inflammatory effects. Despite being classified alongside with polypeptide hormones and growth factors, CKs are distinguishable from them due to some characteristics, such as being produced by a broad range of different cells (stromal cells, fibroblasts, macrophages, B and T cells are few examples) and being pleiotropic, therefore able to act on a remarkable variety of cells and tissue, even sites far from the source of production [1]. CKs bind receptors on the target cells and trigger internal signalling culminating in different events: proliferation, activation, cellular differentiation, apoptosis, and others [2, 3]. CKs production and activation occur during immune perturbation, as infections and inflammatory responses. Inflammation is the natural, physiological reaction to harmful agents, and our main defence against pathogens, irritants and altered cells. Since insults can hit every organ, different body districts can host the inflammatory response: from gut, skin, blood vessels, to compartments that are usually strictly isolated, and protected from external agents, such as the central nervous system (CNS) [4]. When inflammation concerns the CNS, immune cells are recruited to the site of the ongoing injury. In this process, T lymphocytes play a pivotal role: once arrived in the CNS, they interplay with resident cells, mainly neurons and microglia, to fight against the initiating agent. These events stage in a fragile balance between neuroprotection and neuronal harm: abnormal responses driven by CNS-reactive infiltrating T lymphocytes can cause acute and chronic damage, paving the way for pathological conditions. In this case, CNS-infiltrating activated T cells recall other immune cells, produce pro-inflammatory CKs, and activate microglia that in turn reinforces the initial T-cell response, fuelling to damage [5]. Interestingly, beside their well-known role as immunomodulatory agents, accumulating data gathered in the last two decades clearly show that T-cell derived CKs exert neuromodulatory functions in both physiological and pathological conditions acting directly at the level of the synapse [6]. These evidences offer new important insights in our comprehension of neuroinflammatory diseases physiopathology also suggesting unconventional potential targets for treatments development.

In this review, we aim to discuss the mechanisms underlying the interplay between the adaptive immune system and neuroinflammation, with focus on T cells and CKs production mechanisms, and their role in mediating T cells—neurons crosstalk in health and disease including which tools we have today to understand CNS inflammatory mechanisms, from animal models to recent progress in neurophysiological investigations.

## Cytokines as neuromodulators

Neurons are highly specialized excitable cells considered the fundamental effector units of the CNS [7]. This concept has constituted the foundation of experimental and clinical neurosciences since the formulation, in 1888, of the neuron doctrine proposed by Santiago Ramón y Cajal, and it is still offering an invaluable conceptual framework to approach neurobiology [8]. Traditionally, neurons functions have been thought to be regulated specifically by synaptic neurotransmitters release. Decades of investigations supported this view and linked neuronal peculiar electric and secretory activity, together with their organization patterns into complex networks, with fine information processing that enable animal organisms to tune fundamental physiological functions and behaviour in response to environmental stimuli [9, 10]. However, in the last decades, an increasing amount of evidence is highlighting the fact that the modulation of neuronal function is far more complex than previously thought and arise from interrelations between neurons and other cell types rather than from neuronal activity alone [11]. In this framework, the close relationship between the immune system and the nervous system has recently attracted researchers’ attention for its physio-pathological implications. Basing on the classical view of the CNS as an immune-privileged district, the crosstalk between these two systems has been for long intensively disputed [12–14]. Nowadays, the immune-privilege concept has been heavily revised due to the evidence that the immune and the nervous systems share anatomical connections and, more importantly, signalling systems [15–17]. Although the generation of action potentials depends largely on neuronal functional properties and the specific action of classical neurotransmitters, the modulation of synaptic activity has been recently found to be influenced by a wide range of non-neuronal molecular signals.

CKs and chemokines, produced locally by parenchymal glial cells (i.e. astrocytes and microglia) or derived from the peripheral circulation after the crossing of the blood–brain barrier (BBB), have recently shown to exert a fundamental regulatory role in the physiology of healthy CNS, modulating neuron functions [13, 18–20]. For instance, in physiological conditions, interleukin-1β (IL-1β) seems to be required for the maintenance of long-term potentiation (LTP) in the hippocampus, while IL-6 released as a consequence of sustained LTP promotes long term depression (LTD) as a negative feedback mechanism, regulating Hebbian synaptic plasticity implicated in memory processes and in the formation of neuronal circuitry during development [21–23]. Furthermore, several studies showed that glia-derived TNF-α is essential for synaptic scaling, a form of homeostatic plasticity in which the density of postsynaptic neurotransmitters receptors is adapted to match presynaptic activity [24, 25].

These phenomena are mediated by the presence of CKs receptors at the level of synaptic structures, which enable modulating neurotransmitters receptors activity through the activation of intracellular pathways. In this context, the role of IL-1β and TNF-α is perhaps the best characterized from a mechanistic point of view. In CNS excitatory glutamatergic synapses, IL-1 receptor 1 (IL-1R1) is enriched at postsynaptic sites co-localised with NMDA receptors (NMDAR), while TNF-α modulates specifically AMPA receptors (AMPAR) through their association with TNF-α receptor 1 (TNF-R1). CKs receptors activation enables interactions occurring via cytosolic kinase proteins induction, which modulates, through phosphorylation, neurotransmitter receptors activity and trafficking. Particularly, IL-1R1 activation induces NMDAR phosphorylation via Src tyrosine kinase through a MyD88-mediated pathway, while TNF-R1 induces AMPAR phosphorylation via the p38 MAPK pathway. In monoaminergic synapses, a form of homeostatic regulation process involves presynaptic IL-1R and TNF-R1 activation, that lead to increased serotonin transporter (SERT) activity through p38 MAPK-mediated phosphorylation, tuning neurotransmission adjusting serotonin reuptake [23, 26, 27].

## Cytokines in synaptopathy

CKs action on synaptic functions is pleiotropic and dose dependent. Thus, if low levels of proinflammatory cytokines are required for normal CNS function, the dysregulation in their secretion, as seen during many pathological states, can profoundly disrupt neuronal physiology producing a wide range of neurological and psychiatric symptoms mostly caused by synaptopathy (i.e. pathological alterations of synaptic structure and function) that may lead to an excitatory/inhibitory neurotransmission imbalance [21, 23, 28]. For instance, the ability of pathological levels of IL-1β and TNF-α of inhibiting GABAergic currents, weakening the inhibitory transmission by the promotion of GABA_A_ receptors internalization, has been reported by several groups in diverse in vitro model systems [29–32]. On the other hand, a growing body of evidences confirmed CKs ability to perturb neuronal excitability also by the potentiation of excitatory glutamatergic currents in many pathological conditions like epilepsy, hyperalgesia and injury. For instance, in the spinal cord there is an association between TNF-α levels, neuronal sensitization and pain. Particularly, intrathecal administration of TNF-α has been shown to enhance C fibres activity, increasing the response of dorsal horn neurons. Similar CKs effects are also implicated in the excitotoxic phenomena that characterize amyotrophic lateral sclerosis pathogenesis, where motoneurons are more vulnerable to glutamate-induced cell death [26, 33–36].

**Fig. 1:**
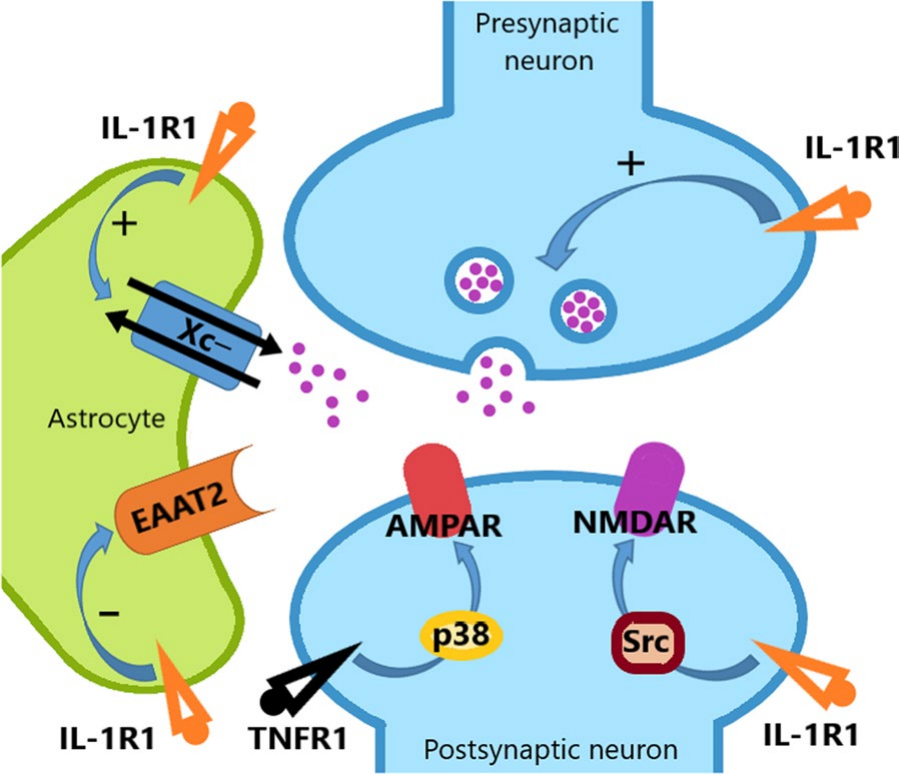
TNFα and IL-1β action at the glutammatergic synapse. Cytokines modulate synaptic function binding to their receptors presynaptically promoting ( +) the release of synaptic vesicles, and postsynaptically modulating receptors activity through phosphorylation. IL-1β is also known to promote astrocytic glutamate release enhancing ( +) the function of Xc^−^ (cystine/glutamate antiporter), while impairing (−) the glutammate reuptake by EAAT2 (mod. form 28)

Importantly, CKs can also hijack synaptic physiology indirectly acting on glial cells. In astrocytes, TNF-α and IL-1β cause an increase in cystine/glutamate antiporter system (Xc^−^) functioning together with a decreased expression and activity of astrocytic glutamate transporters EAATs, resulting in an increased secretion and a decreased astrocytic uptake of glutamate that result in an increased neuronal activity (see Fig. 1) [28, 36, 37]. Furthermore, microglial proinflammatory activation due to altered CKs milieu, as seen in neurodegenerative disorders, can lead to aberrant synaptic pruning and remodelling which can contribute to the excitatory/inhibitory imbalance [38]. Of note, during pathological inflammatory processes, neurotransmitter receptors modulation can also be exerted through the formation of functional complexes with CKs receptors. Interferon-γ (IFN-γ), a proinflammatory cytokine secreted by T cells, is able to induce neuronal dysfunction in cortical neurons enhancing glutamate neurotoxicity through the formation of a neuron-specific, calcium-permeable AMPAR/IFN-γ receptor complex through the binding with AMPAR subunit GluRl [39].

While in classical neurodegenerative diseases, such as proteopathies (i.e. Alzheimer disease, Parkinson disease and amyotrophic lateral sclerosis), the secretion of pro-inflammatory CKs relies mainly on CNS-resident cells, in classical neuroinflammatory autoimmune diseases the major contribution to pro-inflammatory CKs secretion is represented by tissue-invading leukocytes, particularly T lymphocytes [40]. Not surprisingly, in these pathological conditions activated peripheral immune cells infiltrate the CNS parenchyma, causing a profound dysregulation in CKs and chemokines secretion that leads not only to a direct structural damage to neurons by the induction of apoptotic phenomena, but also to a functional synaptic impairment. Multiple sclerosis (MS) represents a prototypical disease of this type. A better understanding of the pathological roles of T cells as a source of CKs in MS can be achieved through an understanding of their physiological functions and their complex relations with the CNS.

## T cells in healthy and inflamed central nervous system

The CNS is a privileged compartment, well protected from external insults thanks to the presence of the BBB. The BBB is a structural and functional barrier, encompassing CNS microvessels, that strictly controls the traffic of molecules from the bloodstream to the CNS. The BBB blocks the passage of pathogens and toxins and controls the traffic of immune cells, ions, and molecules; furthermore, it contributes to CNS functionality and homeostasis [41]. Structurally, the BBB is formed by a basement membrane, the neurovascular unit (NVU), and a complex interplay of endothelial and mural cells, microglia, neurons, and astrocytes [42]. Endothelial cells (ECs) of the BBB are bound by tight junctions forming a continuous barrier, enriched with cadherins, occludin, and claudins [43], whereas astrocytes, pericytes, and components of the extracellular matrix (ECM) surround them, with trophic and structural role [41]. Astrocyte’s foot processes form the glia limitans, a basal lamina that surrounds the blood vessels of the brain and the spinal cord, offering support for neuroglia. When reaching the CNS parenchyma, immune cells migrate also across the glia limitans, mainly thanks to the production of metalloproteinases (MMPs), needed for tissue cleavage [44].

**Fig. 2:**
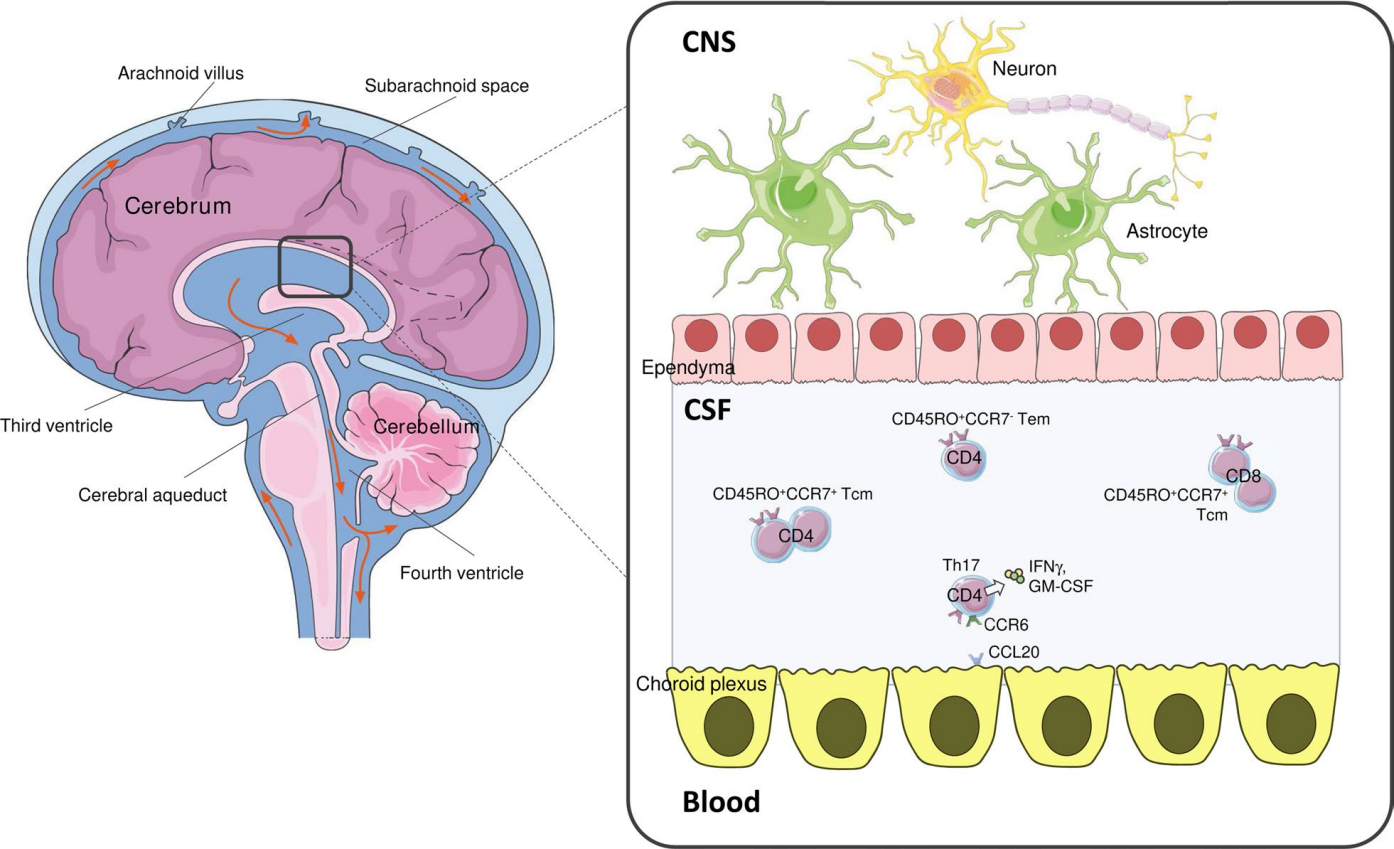
T lymphocytes in the healthy CSF. CSF is produced by ependymal cells in the choroid plexus within brain ventricles and circulates in the subarachnoid space around the brain and spinal cord. CSF provides mechanical protection and supports the flow of nutrients and neurotransmitters. In healthy condition, CSF contains only 1000–3000 cells/mL, including T cells that contribute to immunosurveillance. These are mainly CD4 + , with a ratio of 4:1 versus CD8 + and exhibit a memory phenotype: 90% are CD45RO + CCR7 + central memory T cells (Tcm), and 10% are CDR45RO + CCR7− effector memory T cells (Tem) [47]. Among CD4 + T cells, Th17 are usually present expressing receptors that interact with the choroid plexus, allowing Th17 cell trafficking in the CSF: a main example is CCR6, that binds CCL20 on the choroid plexus [53]. CSF Th17 also may produce low levels of CKs, such as IFN-γ and GM-CSF [52]. Parts of the figure were drawn by using pictures from Servier Medical Art. Servier Medical Art by Servier is licensed under a Creative Commons Attribution 3.0 Unported License (https://creativecommons.org/licenses/by/3.0/)

A healthy BBB usually hampers the passage of large molecules (~ 3000–150,000 Da) and hydrophilic compounds, and allows the traffic of small, hydrophobic and non-polar molecules [45, 46]. In normal conditions, ECs express low levels of adhesion molecules, therefore immune cells migration towards the CNS is abrogated or at least strongly limited; of note, a small fraction of immune cells is present within the cerebrospinal fluid (CSF), that usually contains 1000–3000 cells/mL [47]. T-cell traffic within the CSF mainly occurs in the subarachnoid space and in the choroid plexus, where the CSF is produced. The choroid plexus is characterized by a fenestrated endothelium allowing cell diapedesis, eventually reaching the CNS through the perivascular space [46]. The greatest part (90%) of CSF T cells is represented by CD4 + central memory (Tcm), along with a 10% of effector memory (Tem) [47, 48] (Fig. 2). Their main role is to guarantee CNS immune surveillance [49], as primary defence against pathogens and opportunistic infections, especially in case of immunosuppression [50]. As first characterized by Kivisäkk et al. (2003) [47], the ratio of CD4 + to CD8 + T cells in the healthy CSF is 4 to 1, and most CD4 + T cells show a memory phenotype, expressing markers such as CD45RO, CD27 and CD62LhiCCR7, similarly to CD4 + cells that circulate in peripheral blood. Furthermore, those cells express chemokine receptors as C–C chemokine receptor type 4 (CCR4), CCR5, and CCR6; among these, CCR6 shows a remarkable role for CNS homing of T cells [51]. Few T cells found into the CNS display a T helper 17 (Th17)-like phenotype (CCR6 + CD4 +), produce IFN-γ and granulocyte–macrophage colony-stimulating factor (GM-CSF), and were found particularly enriched in the CSF compared to blood [52].

Of note, the presence of T cells in CNS tissue, beyond their well-known immune patrolling function, seems to have an unexpected role in regulating cognitive functions [53, 54]. Meningeal T-cell populations have demonstrated to be pivotal players in the physiological modulation of behaviour through the secretion of CKs able to influence cortical neurons activity. For instance, several studies showed that meningeal T cells can regulate synaptic plasticity, short term memory and anxiety-like behaviour via IL-17a and IL-4 signalling in neurons [55–57] while T-cell derived IFN-γ is involved in the regulation of social behaviour [54]. Additionally, T-cell depleted mice are characterized by frank cognitive impairments readily restored by passive T cell transfer [58]. Given their role as an important source of neuromodulatory cytokines in physiological condition, not surprisingly, disease-induced dysregulation in T-cell activity and CKs secretion patterns represent a major threat to neuron and synaptic homeostasis.

In pathological conditions, BBB damage allows the migration of active T lymphocytes from the periphery into the CNS. BBB changes during neuroinflammation come along with the increased expression of adhesion molecules that permit T-cell rolling. The P-selectin glycoprotein ligand 1 (PSGL-1), expressed on the surface of T cells, interacts with E/P-selectin on ECs of the BBB [59]. Other involved adhesion molecules are the very late antigen-4 (VLA-4, or α4β1 integrin) and lymphocyte function-associated-1 (LFA-1) [60]. In parallel, activated ECs and astrocytes reduce tightness across BBB junctions during CNS damage, favouring T-cell diapedesis [46].

Our knowledge about neuroinflammation owes much to the study of MS, a human, neuroinflammatory disease, mainly driven by autoreactive T cells that migrate from peripheral blood to the CNS causing demyelination, and its animal model, the Experimental Autoimmune Encephalomyelitis (EAE). The investigation progressed so far allowed to characterize the phenotype of CNS-infiltrating T lymphocytes during neuroinflammation, distinguishing subpopulations and gaining insights into the role of CKs and chemokines.

## T cells and cytokines in MS pathogenesis

MS is an inflammatory, neurological, and autoimmune disease in which T cells are primarily involved. In MS pathogenesis, peripherally activated T lymphocytes migrate across the BBB and infiltrate the CNS, damaging myelin sheath and impairing neuronal transmission. MS approximately affects 3 million people worldwide with a woman versus men ratio of 3:1, respectively, and it is the first cause of neurological disability in young individuals [61]. The antigen triggering the autoreactive response is still unknown; however, MS is considered a multifactorial disease, result of the interplay between genetic and environmental factors [62]: these include human leukocyte antigen (HLA) genes, vitamin D deficiency, individual habits, and infective agents, such as the Epstein-Barr virus (EBV). EBV may be responsible, in susceptible subjects, for the abnormal autoreactive response of T cells against myelin antigens, according to the molecular mimicry hypothesis [63]. Despite not definitively proven as the cause of MS, recent investigations strongly support EBV’s involvement in MS onset [64]. A greater part of our knowledge about MS belongs to the study of EAE. Based on EAE pathogenesis, MS was initially considered driven by CD4 + T cells, mainly Th1 and Th17, according to the well-established involvement of HLA class II haplotypes in MS susceptibility [65]. It was afterward demonstrated that CD8 + T lymphocytes play a fundamental contribution to the disease [66], as they were found in demyelinated lesions; furthermore, CNS-enriched CD8 + express high levels of adhesion molecules, responsible of their rolling through the BBB [67]. CD8 + cells are particularly abundant in cortical MS lesions, commonly reported in severe cases showing quick progression and early development of disability [68].

## T cells and cytokines in EAE

**Fig. 3:**
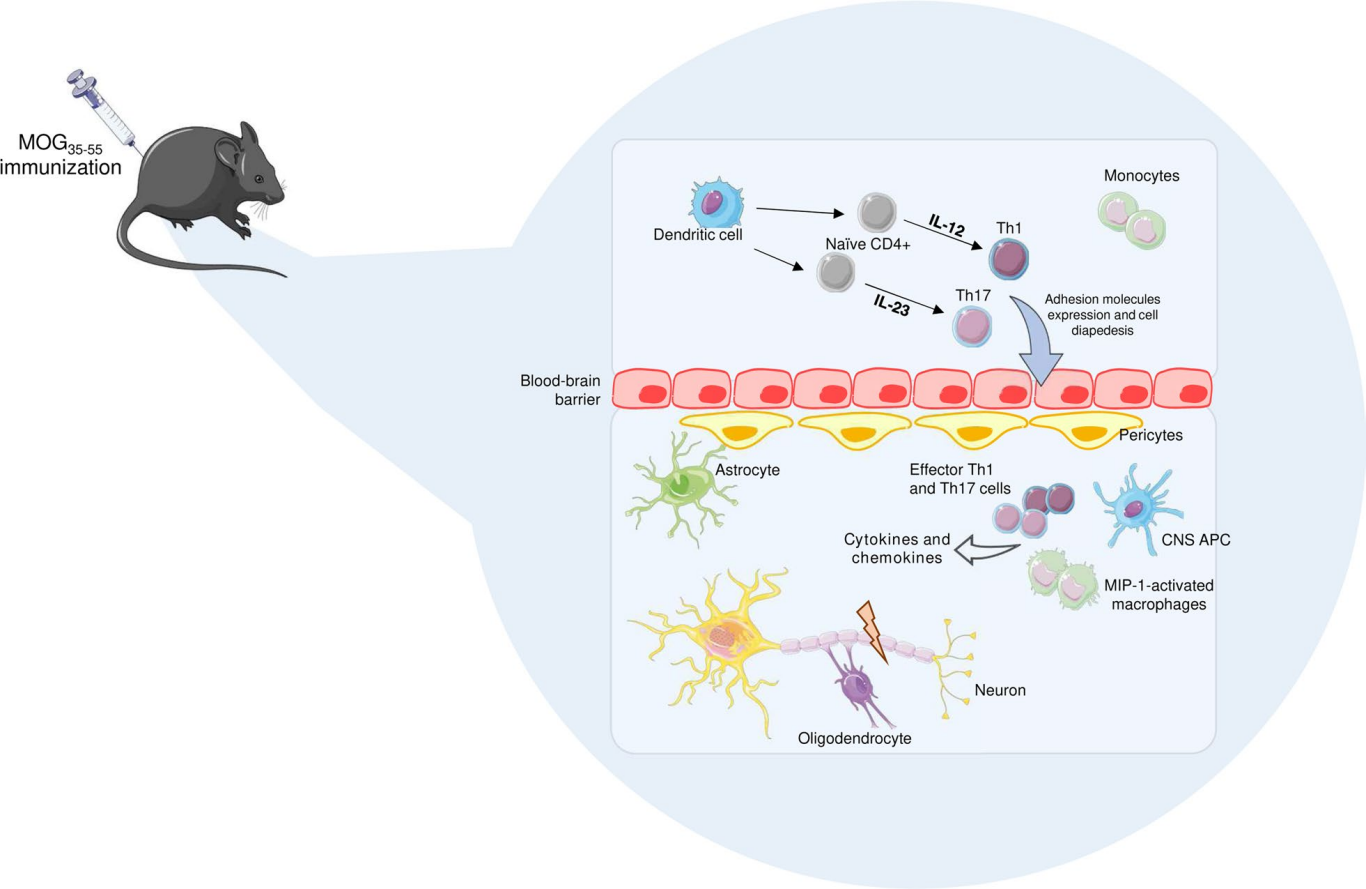
EAE pathogenesis. EAE can be induced in mice by active immunization with encephalitogenic peptide antigen such as the myelin oligodendrocyte glycoprotein 35–55 (MOG_35–55_). Peripheral dendritic cells (DCs) present the antigen to naïve T lymphocytes that are primed to differentiate into T helper 1 (Th1) and Th17 phenotypes, upon interleukin-12 (IL-12) and IL-23, respectively. Monocytes are also recruited and stimulated to differentiate into macrophages upon macrophage inflammatory protein-1 (MIP-1). Activated cells express adhesion molecules and pass through the blood–brain barrier (BBB) endothelium entering the central nervous system (CNS). Local CNS-antigen presenting cells (APCs) reinforce myelin-specific Th1 and Th17 effector and macrophages response. Activated cells produce pro-inflammatory cytokines (CKs) and chemokines that fuel myelin damage [69, 70]. Parts of the figure were drawn by using pictures from Servier Medical Art. Servier Medical Art by Servier is licensed under a Creative Commons Attribution 3.0 Unported License (https://creativecommons.org/licenses/by/3.0/)

EAE represents a reliable animal model of human MS (Fig. 3). EAE is mainly driven by CD4 + T cells, with a secondary contribution of CD8 + cells, and does not spontaneously develop, but requires to be induced by active immunization with encephalitogenic antigens, such as the myelin oligodendrocyte glycoprotein 35–55 (MOG_35–55_), and the proteolipid protein 135–151 (PLP_135-151_), emulsified in complete Freund's adjuvant (CFA) [69, 70]. Less commonly, EAE can be induced by adoptive transfer of CD4 + T cells reactive against CNS self-antigens into naïve animals [71]. In EAE, autoreactive CD4 + T cells differentiate into T helper 1 (Th1) and Th17 within secondary lymphoid organs and migrate from peripheral blood into the CNS parenchyma mainly through the choroid plexus, leading to cellular infiltrates, demyelination, and axonal damage [72, 73]. T-cell diapedesis through the BBB occurs at parenchymal ECs level and within leptomeningeal spaces, where activated T cells recognize CNS autoantigens carried by local antigen presenting cells (APCs) [74, 75].

Th17 cells produce pro-inflammatory CKs, first IL-17. These cells differentiate from Th in response to the transforming growth factor β (TGFβ), IL-1β and IL-6, and are sustained in proliferation and activation by IL-23 [76]. Along with Th17, pro-inflammatory Th1 cells producing IFN-γ contribute to CNS damage in EAE [77]. T-cell rolling through the BBB occurs as immune surveillance in normal conditions, as abovementioned, but increases during neuroinflammation, allowing the migration of pathogenic cells. The study of EAE showed that Th1 cells entrance in the spinal cord of mice is mediated by LFA-1 (or αLβ2 integrin) interacting with adhesion molecule 1 (ICAM-1) [74], along with the binding between CCR7 on T cells and CCL19/CCL21 on the BBB, as well as within draining lymph nodes [78, 79]. This interaction is fundamental to allow T-cell migration into the CNS, as confirmed by the high concentration (90%) of CCR7 + T lymphocytes in the human CSF [47]. Furthermore, T-cell interaction with BBB components is supported and mediated by G-protein-coupled receptors (GPCRs) [80]: during neuroinflammation, P-selectin within CNS microvasculature undergoes upregulation and interacts with PSGL-1 expressed on T cells [59]. In this context, GPCRs signalling allows T-cell arrest on the BBB by regulating integrins (ICAM-1, VLA-4, etc.) expression and activation [81]. Such mechanisms are associated with the migration of either Th1 and Th17 into the CNS in in vitro and in vivo models; interestingly, the selective deletion of T-cell expressed α4 integrin delays the onset of EAE and drives Th1 and Th17 migration towards different regions of the CNS: mice lacking α4 integrins showed infiltrates of Th1 in both spinal cord and brain, whereas Th17 were present only in the brain [82].

The complex balance between Th1 and Th17 in EAE has been widely investigated in the last decades, unravelling important mechanisms in disease pathogenesis. Ferber et al. demonstrated in 1996 that the abrogation of IFN-γ in mice determined an aggravation of EAE severity, in contrast to the initial hypothesis that IFN-γ produced by Th1 cells was necessary to develop EAE [83]. In the same year, similar results were obtained in mice deficient for IFN-γ R [84]. Later studies gained insights into these controversial findings concerning IFN-γ in EAE reporting that, beyond the pro-inflammatory effect, this CK might contribute to clean the CNS from myelin debris and reduce oxidative stress damage [85]. Furthermore, IFN-γ is required for the expression of the programmed death-ligand 1 (PD-L1), expressed on many cell types, that supports T-cell homeostasis, prevents abnormal immune responses and autoimmunity [86]. It was later found that IL-12 and IL-23 share the p40 subunit [87] and that EAE mice lacking IL-12p40 expression were resistant to the disease development [88, 89]. This suggested that IL-23 is crucial for Th17 differentiation and, consequently, EAE susceptibility [90, 91]. Th17 differentiation in EAE is also sustained by IL-1β and IL-21, critical for IL-1R signalling and IL-23R expression [92, 93]. Interestingly, recent findings concerning IL-12 showed that, despite generally being a pro-inflammatory CK, it can exert a neuroprotective role in EAE, since the ablation of its receptor in neurons and oligodendrocytes of mice leads to a more severe inflammation compared to controls; furthermore, IL-12R has a similar distribution pattern in the brain of MS patients and EAE mice [94].

In 2001, McQualter et al. showed that GM-CSF-deficient mice are resistant to EAE. These animals did not display immune cell infiltrates and demyelination at central level, suggesting that GM-CSF is critical for immune cells migration into the CNS [95]. This was later confirmed by studies demonstrating that GM-CSF is required for microglial maturation and CCR2 + monocytes recruitment into the CNS [96, 97]. One year later, it was showed that TNF-α is also involved in EAE development and severity, since TNF-α-deficient mice reported a delayed onset and a reduction in the expression of different CKs and chemokines; that was remarkably true for CCL5, and macrophage inflammatory protein-1α (MIP-1α), also called CCL3, both fundamental for cells recruitment in the site of inflammation [98]. These findings suggested that TNF-α could act synergically with GM-CSF and IL-17 for cytokines and chemokines production during EAE development.

## Cytokine-induced synaptopathy in MS and EAE

During the last two decades, accumulating findings have indicated synaptopathy as a hallmark of MS pathogenesis [99]. Histochemical analysis clearly showed a substantial synaptic loss with a reduction in synaptic spine density and dendritic length in samples derived from both MS patients and EAE rodents. These histological findings have been also correlated with a significant reduction in the tissue levels of proteins with crucial roles in synaptic maintenance and function (i.e. neurexin–neuroligin complex, synaptophysin and synaptotagmin). Interestingly, low levels of synaptophysin have been found also in non-demyelinated brain regions, suggesting that synaptopathy is an early event in MS pathogenesis, independent from demyelination and grey matter damage, while shows a strong correlation with the altered levels of CKs in the CNS tissue [100, 101]. Noteworthy, these processes equally affect glutamatergic and GABAergic synapses suggesting an important role of excitatory and inhibitory neurotransmission in the genesis of MS symptomatology, giving reasons of the cognitive impairment that often precede motor symptoms [102, 103].

Structural changes are accompanied by functional alterations. Electrophysiological investigations on EAE mice-derived acute brain slices, found significant increase in AMPAR-mediated glutamatergic neurotransmission correlated with activated microglia and high levels of TNF-α. These phenomena were blocked by the inhibition of TNF-α signalling confirming a strong relation between this CK and glutamatergic neurotransmission modulation. Notably, according to the early structural synaptic alterations found in non-demyelinated brain regions, microglia activation and changes in the expression and functional properties of glutamate AMPAR preceded the symptomatic phase in EAE mice [104]. These changes are consistent with the findings of activated microglial nodules in MS pre-active lesions where these cells can be found in close proximity with axon terminals [105], while in active lesions microglia and macrophages have been found to actively remove spines characterized by local calcium overload after pathological excitatory inputs [106].

An interesting study performed by exposing mice brain slices to MS patients’ cerebrospinal fluid revealed that transient receptor potential vanilloid 1 (TRPV1), a cation channel known to facilitate glutamatergic neurotransmission, is essential in mediating NMDAR-mediated neurotransmission and excitotoxicity induced by IL-1β during the chronic phase of the disease [107]. Intriguingly, the same group also show that TRPV1 ablation results in higher lethality of EAE mice in the peak phase of the disease and a better recovery of the surviving animals in the chronic stages. These apparently contradictory results can be explained by the fact that the peak phase of the disease is mainly driven by the effect of TNF-α while during the chronic stages IL-1β seems to take the centre stage. Electrophysiological recordings confirmed that the absence of TRPV1 enhances glutamate-mediated neurotransmission, described at the peak phase of EAE, while it dampens the decrease in GABAergic synapses activity at the chronic phase [108].

Experiments investigating the mechanisms responsible for cognitive deficits in MS, showed impairment in the induction of hippocampal long-term potentiation (LTP, the electrophysiological correlate of memory formation functions) associated with an altered NMDAR homeostasis, an increase in the hippocampal microglial infiltration and high levels of IL-1β [99]. IL-1β is also highly expressed in EAE mice cerebellum as a consequence of infiltrating lymphocytes release. Electrophysiological investigations and biochemical analysis revealed an excitatory/inhibitory dysregulation in balancing synaptic transmission at the level of Purkinje cells. The glutamatergic transmission was enhanced due to glial involvement: a reduced expression and functioning of glutamate aspartate transporter EAAT1 expressed by Bergmann glia (i.e. cerebellar astrocytes) was found [99].

Beside the classical activated T CD8^+^ lymphocytes Th1 oriented, it is known that in EAE neuroinflammation, IL-17 produced by γδ T cells contributes to disease development and pathological responses [109]. As other CKs in the brain, IL-17 participates in healthy neurophysiological processes. Recently, Miguel Ribeiro and colleagues showed that mice lacking γδ T cells or IL-17 displayed deficient short-term memory, with normal long term one. Authors hypothesis is that γδ T cells might contribute by producing IL-17 in the brain meninges, without penetrating the brain parenchyma, and increase glutamatergic synaptic plasticity of hippocampal neurons possibly by promoting brain-derived neurotropic factor (BDNF) production from glial cells [56]. Indeed, it has been demonstrated that astrocytes and microglial cells express IL-17RA and respond to IL-17 treatment in a dose dependent manner [110]. For this reason and due to their anatomical distribution, these cells may sense meningeal molecular environment, including CKs, allowing a bidirectional crosstalk with the brain and spinal cord parenchyma modulating neurons functions in health and disease. For instance, the effect of IL-17A on astrocytes uptake of glutamate has been investigated and results show that low concentrations (10–50 ng/mL) of IL-17A may promote neuronal excitotoxicity by impairing glutamate uptake by astrocyte, whereas high concentrations (~ 100 ng/mL) did not modify glutamate transporter expression. These apparently contradictory data can be explained by the hypothesis that at higher doses IL-17 is able to also stimulate IL-17D receptor that could act as a negative feedback system blocking the downstream cascade of IL-17A receptor [111].

Besides its action on glial cells, IL-17 may also have a direct impact on neurons, and IL-17R expression has been reported in neural cells [112]. In dorsal root ganglion (DRG), IL-17 is implicated in pain regulation: it was found to regulate inflammatory responses associated with neuropathic pain induced by nerve injury, and IL-17R was detected in DRG. IL-17 produced by spinal cord astrocytes may play a role in inflammatory pain [113], and physiological level of IL-17 directly increases interneuron responsiveness to presynaptic input [114]. Recently, Hao Luo and colleagues described how IL-17 signaling contributes to allodynia and hyperalgesia in spinal cord neurons and gave insight into neuron-glia interactions where IL-17 produced by astrocytes enhances neuronal activities and promote neuropathic pain. Authors showed that IL-17 and IL-17R act through multiple mechanisms: neuronal-glial interactions and central and peripheral sensitization; in the spinal cord IL-17 enhances NMDA-receptor-mediated currents and suppresses inhibitory GABA_A_-receptor mediated currents [115]. Furthermore, a recent work from Massimiliano Di Filippo and colleagues showed that IL-17A is able to disrupt hippocampal synaptic plasticity in dose-dependent manner in EAE mice, blocking LTP. Similar to TNF-α and IL-1β, the negative effects exerted by IL-17AR activation on LTP induction were found to be mediated by the modulation of the kinase p38 MAPK. Indeed, brain slices treatment with IL-17A resulted in the phosphorylation of this kinase, while, on the other hand, the pharmacological inhibition of p38 MAPK was able to reverse the synaptic alteration induced by this cytokine [116].

Historically, the mechanistic study of MS molecular pathogenesis has dedicated much attention to classical cytokines. For this reason, the role of GM-CSF in the activation of microglia and its effects on neuron function, survival, synaptic transmission and excitability, is still mostly unknown. CKs in the brain may cause, even in the absence of invading T cells, inflammatory neurodegeneration or direct alteration of neuron function, hyperexcitability, without observing an increase of the inflammatory cytokine release. These effects are mediated by the variable modalities by which microglia activate and interact with other glial cells and functional neurons. A recent study on chronic GM-CSF exposure in organotypic hippocampal cultures showed altered neuronal electrophysiological properties in the absence of inflammatory neurodegeneration, but with the occurrence of microgliosis. In this work, GM-CSF affected neuronal networks gamma oscillations, a pattern of neural activity correlated to cognitive processes detected in diverse brain regions. Furthermore, GM-CSF induced microglia proliferation, yet in the complete absence of inflammation and neurodegeneration. The neurophysiological consequence of this activation was the induction, in a dose dependent fashion, of altered network patterns [117].

In MS, GM-CSF interacts with IL-17 during neuronal alteration exacerbations, but IL-17 alone is not affecting the gamma oscillations that is a specific GM-CSF effect microglia dependent [118, 119].

Recently, we investigated the electrophysiological effects of a CKs cocktail, containing GM-CSF (TNF-α, IL-1β and GM-CSF). This CKs mix, which was intended to mimic a MS-like inflammatory microenvironment, was used to treat organotypic spinal slice culture from mice embryos, an in vitro culture where sensory-motor cytoarchitecture, synaptic properties and spinal resident cells are retained in a 3D tissue organization. In these cultures, we monitored the emergence of synaptopathology in pre-motor circuits following CKs transient exposure. By patch-clamping ventral interneurons, we measured a significant increase in spontaneous synaptic activity due to CKs treatments characterized by a speeding up of the decay phase of GABAergic inhibitory currents. Such a protocol allowed unmasking subtle early changes in GABAergic synaptic currents, which are significant players in excitation/inhibition network balance of premotor circuits. These changes in electrical activity were accompanied by a significant, endogenous, production of CKs and chemokines with astrogliosis and microglia activation. In this model we investigated possible neuro-protective strategies to address synaptic changes during chronic CNS inflammation [120]. In a further study, we investigated to what extent the tuning of GABAergic currents duration is a general response to any local alteration of the inflammatory status in the spinal cord. We found that only the CKs cocktail, containing GM-CSF, promotes changes in inhibitory transmission time course, probably due to the increase expression of GABA_A_ receptor α-subunit. This mechanism might be a targetable pathway in spinal cord inflammatory treatments [31].

## Recall chemokines in MS

CSF represents a site of election for the study of MS pathogenesis as well as in supporting diagnosis at disease onset [121]. In case of suspect MS, CSF is withdrawn by lumbar puncture and investigated for the presence of oligoclonal bands (OCBs) of intrathecal immunoglobulins (Ig), considered a hallmark of the disease. OCBs produced by B lymphocytes undergo clonal expansion after the encounter with an unknown antigen. Given that OCBs are not exclusive of MS, but also common to other neurological diseases, i.e. autoimmune encephalitis [122], their detection must be sided by supporting findings collected by other laboratory analysis, MRI, and clinical evaluation of the patient. Furthermore, CSF is usually investigated with paired serum samples, since OCBs patterns are meaningful of differential diagnosis: MS patients generally report unmatched OCBs, present in the CSF but not in the serum [123, 124]. The importance of B cells in MS onset and their involvement in OCBs production is reinforced by studies reporting increased levels of CXCL13, a strong chemoattractant for B cells produced by APCs [125], in the CSF of MS patients [126]. For this reason, CXCL13 has been suggested as a potential biomarker of the disease. In addition, Khademi et al. correlated the increased levels of CXCL13 with relapse rate, Expanded Disability Status Scale (EDSS) value and number of MRI lesions. Recent findings confirmed that CXCL13 index might be a good predictor of future disease activity in MS patients, alongside other markers of neurological damage such as neurofilament light (NfL) [127]. 

On the same line, other recall chemokines have been found upregulated in the CSF of MS patients, as further demonstration of the crucial pathogenic role of CNS-infiltrating immune cells upon MS onset and acute phase. CXCL9 and CXCL10 are two classical recall chemokines responsible for cell infiltration, specifically through the interaction with the CXCR3 receptor [128]. CXCL9, also called monokine induced by gamma interferon (MIG), is chemotactic for effector T cells (mainly Th1), macrophages and natural killer cells (NK). CXCL10 attracts various immune cells like T cells and NK [128]. 

CCL17 and CCL22 are two recall chemokines that interact with the same receptor, CCR4, expressed on activated T cells, including Th1, Th2 and Th17 phenotypes [130]. Of note, CCR4 is also expressed on murine dendritic cells (DCs) and necessary for EAE development, as it would sustain Th17 cells by GM-CSF and IL-23 production [131]. Elevated levels of CCL17 and CCL22 have been found in the CSF of MS patients, supporting their role in strongly promoting T-cell infiltration into the CNS [132]. Interestingly, CCL22 may sustain MS development not only by allowing cells infiltration, but also being locally expressed: this chemokine has been found expressed by CNS activated microglia within demyelinated lesions, remyelinated areas, as well as in normal-appearing white matter, supporting the hypothesis of a potential switch across microglial phenotypes that may be predictive of incoming demyelination or remyelination processes [133, 134].

CCL3 and CCL4, also known as macrophage inflammatory protein 1-α and 1-β (MIP 1-α and MIP 1-β), are two recall chemokines that interact with CCR5 (both) and CCR1 and CCR4 (CCL3) and are responsible for the recruitment of polymorphonuclear leukocytes (especially CCL3), NK and monocytes (mainly CCL4), as well as of activated T cells [135]. Already in 1998, the expression of these two chemokines was investigated in MS patients and found increased in *post-mortem* lesions, expressed either locally or by infiltrating macrophages [136]. Few years later, in 2002, Mahad et al. investigated several recall chemokines in MS CSF compared to patients with benign headache, with non-inflammatory neurological diseases (NIND), or with other inflammatory neurological diseases (IND). The study reported an increase of CXCL10 and a decrease of CCL2, also known as monocyte chemoattractant protein 1 (MCP1), during relapse, whereas levels of CCL3, CCL4, and CCL5 did not significantly differ among groups [137]. Authors discussed the reduced levels of CCL2 within the CSF by suggesting its sequestration and localization within the CNS right after clinical onset. In recent times, both CCL3 and CCL4 have been found elevated into the CSF of Relapsing–Remitting MS (RRMS) patients, and positively correlated with disease duration and severity, measured by EDSS [135]. Authors therefore suggested these two chemokines as predictive biomarkers for disease severity and potential therapeutic targets.

Concerning CCL2, data are generally controversial. CCL2 attracts T cells, NK, DCs, and monocytes by interacting with CCR2. The block of CCL2-CCR2 interaction in EAE only slightly limited relapses and reduced macrophage recruitment within the CNS, as well as Th1 response [138, 139]. In human MS, CCL2 appears even reduced in the CSF, contrarily to other chemokines that are increased, as abovementioned [137, 140]. Recently, Sørensen et al. showed that CCL2 levels are similar in the CSF of MS compared to optic neuritis patients and in peripheral monocytes of MS compared to healthy controls, suggesting that this chemokine may have a limited role in MS pathogenesis [141]. Recall chemokines involved in MS are recapitulated in Table 1.

**Table 1:** Recall chemokines involved in MS pathogenesis

Chemokine	Receptor	Function	Role in MS
CXCL9	CXCR3	Recall for Th1, macrophages, NK	Cell infiltration [128]
CXCL10	CXCR3	Recall for monocytes, macrophages, T cells, DCs, NK	Cell infiltration [128, 129]
CXCL13	CXCR5	B-cell chemoattractant	CSF OCBs production [125–127]
CCL2	CCR2	Recall for T cells, NK, DCs, monocytes	Controversial; reduced in MS CSF [137, 140]; similar levels between MS, optic neuritis, and peripheral monocytes of healthy controls [141]
CCL3	CCR1, CCR4	Polymorphonuclear leukocytes chemoattraction	Cell infiltration; increased in RRMS CSF [135]
CCL4	CCR5	Recall for NK and monocytes	Cell infiltration; increased in RRMS CSF [135]
CCL17	CCR4	Th cells recall	T-cell infiltration [132]
CCL22	CCR4	Th cells recall	Cell infiltration and activation of CNS-resident microglia [133, 134]

*DCs* dendritic cells, *CSF* cerebrospinal fluid, *NK* natural killer, *OCBs* oligoclonal bands

## Pro-inflammatory cytokines in MS and potential as therapeutic targets

As discussed above, we know from EAE study that pro-inflammatory CKs produced by pathogenic T cells fuel inflammation, immune cells recruitment and CNS damage also in human MS. Some of these CKs have a critical role in disease pathogenesis and have been widely investigated (the role of CKs in MS is summarized in Table 2).

**Table 2:** Pro- and anti-inflammatory cytokines involved in MS pathogenesis and therapies

Cytokine	Producer	Function	Role in MS	Therapies
IL-1α, 1β	DCs, NK, B cells, monocytes/macrophages, fibroblasts, etc	Pro-inflammatory: produced by dying cells (IL-1α) and inflammasome (IL-1β), both initiate inflammation [196]	Correlation with number and volume of cortical lesions and disease severity, especially IL-1β [167, 168]	Not reported
IL-4	Mast cells, eosinophils, basophils,	Immunomodulatory; Th2 differentiation; IgE production; tissue healing [190]	Possible correlation between IL4 gene polymorphism and MS susceptibility [194]; increased serum levels in MS and NMO [195]	Not reported
IL-6	T and B cells, macrophages	Pro-inflammatory; response to infection and tissue damage [160]	Elevated in MS CSF during acute phase[161]; correlation with disease severity [162]	Probably reduced upon GA treatment [160]
IL-10	Monocytes, Th2, Tregs, B cells, mast cells	Anti-inflammatory	Decreased in MS plasma [185]; decrease in CSF was correlated to worsened MS progression [187]	Modulated by ATX-MS-1467, mixture of MBP, in preclinical studies and MS ongoing trials [188, 189]
IL-12/IL-23	DCs	Generally pro-inflammatory; recent findings highlighted a possible neuroprotective role [94]; Th17 differentiation	SNPs-dependent increased MS susceptibility [150]	Ustekinumab (anti-IL-12/23p40) failed phase II trial on RRMS [151]
IL-17	Th17	Pro-inflammatory; pathogens response, soluble factors production [142]	Increased IL-17 mRNA in CSF and IL-17 level in CNS lesions [148]; increased Th17 in MS CSF during exacerbations [149]	Ref. ustekinumab in IL-12/IL-23
GM-CSF	Macrophages, mast cells, NK, T cells, fibroblasts, etc	Cell differentiation and activation against pathogens and tissue damage [156]	Increase of GM-CSF-producing memory T cells in MS patients [157]	MOR103 (anti-GM-CSF) was well tolerated during phase 1b trial on SPMS and RRMS [158]. Ocrelizumab may improve MS course also by depleting GM-CSF-producing B cells [159]
IFN-β	Monocytes, fibroblasts, endothelial cells, etc	Viruses response and defense; antigen-presentation induction; activation of NK and macrophages [174]	Mainly studied in EAE: IFN‐β − / − mice developed earlier and severe EAE [175, 176]	IFN-β1 is the main first-line RRMS treatment [179], acting by modulating T-cell differentiation, DCs migration and CKs production [181]
IFN-γ	Activated B and T cells, mainly Th1	Pro-inflammatory; activation of T, B cells and macrophages	Controversial: IFNγ administration worsened MS [152], but anti-IFNγ aggravated EAE in mice [154]. Possible protective role towards mature oligodendrocytes [155]	Not reported

IL-17 is produced by Th17 cells either in EAE and MS. It is a pro-inflammatory CK primarily responsible of anti-microbial defence in various tissues, and stimulates the production of other inflammatory factors, including CKs, chemokines, and metalloproteinases [142, 143]. Th17 differentiate from Th1 cells upon involvement of transcriptional factors (i.e. JAK-STAT3 axis, followed by RORγτ and RORα activation) and stimulation by CKs like IL-23, IL-1β, IL-6, IL-21, and TGFβ [144]. On the other hand, Th17 may differentiate directly from naïve CD4 + T cells upon stimulation by IL-23, TGFβ1 and IL-6 [145]. As abovementioned, IL-23 shares the p40 subunit with IL-12 and it is known that EAE mice that do not express IL-12p40 do not develop the disease [87, 88]. The importance of Th17 in EAE fuelled the interest on the role of this T-cell subpopulation, and consequently their mainly produced CKs IL-17, in MS patients [146]. IL-17 mRNA resulted elevated in the CSF and peripheral blood of MS patients; interestingly, levels were higher in peripheral blood during disease relapses. Furthermore, IL-17 was found in CNS lesions [147, 148]. Analysis of T-cell phenotype in MS CSF and blood showed that Th1 are generally more abundant, compared to Th17, in both compartments, suggesting that Th17 may have a critical role into the CNS rather than periphery [149]. Th17 were also found increased in CSF during MS exacerbations, whereas Th1 did not significantly change [149]. Findings pointing out Th17 relevance in MS pathogenesis have been reinforced by genetic investigations on single-nucleotide polymorphisms (SNPs) of IL12B (encoding for IL-12p40) and IL-23 receptor (IL23R) genes, identifying few specific SNPs (i.e. IL23R rs11209026) associated with increased MS susceptibility [150]. The proven importance of IL-17 and the IL-12/IL-23 axis paved the way for investigating the effectiveness of an anti-IL-12/23p40 antibody, ustekinumab, on RRMS patients. A phase II trial was conducted on 249 RRMS, treated (or not) by repeated subcutaneous injections of ustekinumab [151]. Unfortunately, the antibody did not pass the trial: patients did not show any significant clinical or radiological improvement, and 3% of them reported severe adverse events.

IFN-γ is a pro-inflammatory CK produced by Th1, therefore of great interest in MS investigations. Panitch et al., in 1987, showed that IFN-γ administration to MS patients caused a worsening of the disease, with increased relapses [152]. On the other hand, later studies suggested that IFN-γ may also have a regulating role limiting MS severity, as EAE mice treated with anti-IFN-γ reported aggravated disease [153, 154]. Lin et al. discussed that IFN-γ would exert a protective role towards oligodendrocytes, promoting their survival, and that IFN-γ’s beneficial or detrimental effect in neuroinflammation might depend on administration timing. Specifically, IFN-γ would be beneficial for mature oligodendrocytes that have already produced myelin in early stages, but damaging for remyelinating oligodendrocytes that show higher sensitivity to cell stress [155]. Authors suggested that IFN-γ administration before EAE onset would improve the disease. However, despite intriguing results, to date IFN-γ is not considered a target for MS treatment.

As mentioned, mice lacking GM-CSF do not develop EAE, mainly because immune cells fail to migrate into the CNS [95]. GM-CSF is a glycoprotein produced by various type of cells (macrophages, mast cells, NK, T cells, fibroblasts, and so on) that stimulates granulocytes and monocytes differentiation and activation against infections and in inflammatory processes [156]. In MS patients, GM-CSF-producing memory T cells are increased [157]. A randomized phase 1b trial in 2015 was performed by intravenously administering a human anti-GM-CSF antibody, MOR103, to RRMS and Secondary-Progressive MS (SPMS) patients [158]. The treatment was overall well-tolerated and reported low immunogenic potential. Interestingly, a study conducted on MS patients treated with ocrelizumab, an anti-CD20 monoclonal antibody, showed that the depletion of GM-CSF-producing B cells correlates with improved MS course, suggesting their pathogenic role in the disease [159].

IL-6 is produced by T, B cells and macrophages and stimulates the response against infections and tissue damage. It binds to IL-6R activating different molecular pathways, like JAK-STAT and mitogen activated protein kinase (MAPK), culminating with the production of pro-inflammatory factors involved in inflammatory responses [160]. IL-6 concentration was found to be increased in MS plasma and CSF during acute phase and decreased upon recovery [161]. Recent studies gained insight into IL-6 fluctuations in the CSF of MS patients, showing that its level undergoes an increase in similar proportion among different MS courses and that it correlates with clinical and radiological signs of severe disease [162]. To date, IL-6 is not tested as a specific target of MS treatments. However, it is believed that the effectiveness of immunomodulatory drugs currently used in MS may depend, at least in part, on the reduction of pro-inflammatory CKs, such as IL-6. This is the case of Glatiramer acetate (GA), a polymer composed of four amino acids in common with myelin basic protein (MBP). The exact mechanism of action of this drug is still partially unclear, but it seems able to drive T-cell response against itself instead of myelin and to shift T-cell phenotypes from pro-inflammatory Th1 to Th2 [163], with consequent decrease of circulating pro-inflammatory CKs-producing T cells [160].

Among pro-inflammatory CKs, it is worthwhile to mention the IL-1 family and TNF-α. Main components of the first are IL-1α and IL-1β, generally induced upon stimulation by various immune cells, and classically involved inflammatory pathways. Both bind the receptor complex IL-1R1 and IL-1RAcP, triggering a cascade of events involving MyD88 signalling, activation of the nuclear factor kappa-light-chain-enhancer of activated B cells (NF-κB), and so on [163, 164]. Mice lacking IL-1R and IL-1β are resistant to EAE induction [165, 166]. Contrarily, less is known about IL-1α. In MS patients, IL-1β in MS CSF was correlated with cortical lesions number and volume [167] and higher levels of IL-1β were found in the CSF of MS patients with severe disease [168]. In general, IL-1β has been suggested as able to stimulate autoreactive T-cell response and production of pro-inflammatory CKs, with a critical contribution to the formation of CNS lesions; however, its role in MS is still unclear [169].

Finally, TNF-α is a CK with multiple functions and involved in the pathogenesis of several inflammatory and autoimmune diseases. It binds receptors TNFR1 and TNFR2, that are expressed on most cell types or on epithelial, endothelial, and immune cells, respectively [170]. TNF-α is produced by various cell types, from macrophages to lymphoid cells, neurons, mast cells, endothelial cells and so on. Its main function is promoting inflammatory responses by activating signalling such as MAPK and NF-κB [169]. In MS, TNF-α can exert opposite effects on CNS, detrimental for neurons and axons or protective; it was found increased in CSF and in brain lesions of patients [171, 172]. Once established the emerging role of TNF-α in MS pathogenesis, the interest of clinical research shifted on TNF-α inhibitors, or blockers. Some of them are FDA approved for the treatment of autoimmune and inflammatory diseases, i.e. infliximab (Crohn's disease, psoriasis, Rheumatoid Arthritis, etc.) and golimumab (ankylosing spondylitis, Rheumatoid Arthritis, and others) [172]. However, TNF-α blockers were considered unsafe for MS, since treated patients reported demyelinating events [171]. Studies suggested that this detrimental effect may be due to the non-specific TNF-α blockers mechanism, acting simultaneously on the receptors TNFR1 and TNFR2; through TNFR2 binding, TNF-α usually exerts a neuroprotective effect that would be suppressed by TNF-α inhibitors increasing susceptibility to demyelination and axonal damage [173].

## Anti-inflammatory cytokines

Literature reports interesting findings about anti-inflammatory CKs in MS. Interferon-β (IFN-β) is a protein encoded by the *IFNB1* gene that belongs to the type I class of IFNs; it is involved in pathogens response, especially in fighting viral infections [174]. IFN-β has shown the capability to modulate neuroinflammation through various mechanisms. IFN‐β − / − mice developed earlier EAE onset and progressed faster, with aggravated neurological damage compared to IFN‐β + / + mice [175, 176]. Pennell and Fish enlightened the mechanism behind the IFNβ immunomodulatory effect in EAE by showing that IFN‐β − / − mice were characterized by highly activated DCs that stimulated T-cell differentiation into pathogenic Th17 phenotype and that were prone to rapidly migrate into the CNS [177]. In another investigation, Wang et al. showed that IFN-β induced CD25 + FOXP3 + Tregs in EAE C57BL/6 mice in neuroantigen-dependent manner, by incorporating IFN-β and neuroantigen in the Alum adjuvant as tolerogenic vaccination [178]. The immunomodulatory potential of IFN-β has been successfully translated to human MS since 1993, when IFN-β1 was approved as first-line RRMS treatment [179]. Patients treated with IFN-β1 exhibited reduced relapse rate, slowed disease and disability progression, and decreased lesions number [180]. Interestingly, a recent study reported a significant decrease in serum concentration of IL-1β, IL-12/IL-23p40 and IL-18 in 30 IFN-β1-treated RRMS compared to naïve patients, suggesting that IFN-β1 may act synergically on multiple levels, i.e. modulating T-cell differentiation, DCs migration, and pro- and anti-inflammatory CKs production [181]. Concerning anti-inflammatory CKs, a few years before D'Angelo et al. investigation, Zhang et al. showed that IFN-β was able to reduce Th17 proliferation and increase the production of IL-10 in T cells stimulated in vitro [182]. IL-10 is a classical anti-inflammatory CK encoded by the IL10 gene in humans and produced by monocytes, Th2 lymphocytes, Tregs, B cells, and mast cells; it mainly acts regulating the JAK-STAT pathway and blocking NF-κB activity [183]. IL-10 − / − mice developed aggravated EAE compared to IL-10 + / + mice; furthermore, peripheral T cells of mice lacking IL-10 expression produced higher levels of pro-inflammatory CKs and were able to induce severe EAE in wild-type mice upon transfer [184]. Reduced plasma level of IL-10 in MS patients has been already documented in the late 90’s [185]; this decrease appeared abrogated during IFN-β1 treatment, particularly in responder patients [186]. Of note, a 2022 study on 106 RRMS patients reported correlation between fatigue, worse disease progression and decreased IL-10 in CSF [187]. Concerning treatments, the drug ATX-MS-1467, a four-peptides mixture of human MBP, showed interesting potential as immunomodulant, in part through IL-10 modulation, as reported in preclinical studies on EAE and in trials on MS patients [188, 189]. However, trials are still ongoing, and its mechanism of action has yet to be clarified.

Little is known about IL-4. It is produced by mast cells, eosinophils, basophils, and has a critical role in the differentiation of naïve T cells into Th2. Th2 cells, in turn, produce IL-4, which stimulates them in a positive-feedback loop mechanism [190]. Data about IL-4 role in EAE are controversial: despite findings showing the importance of Th2 and IL-4 in EAE development [191], it has been documented either a worsening or not of EAE progression in mice lacking IL-4, probably depending on animal strain and genetic background [192, 193]. In humans, it has been suggested an increased MS susceptibility based on IL4 gene polymorphism [194] and elevated serum level of IL-4 in neuromyelitis optica and MS, but without any significant difference between them [195].

## Central nervous system-resident T cells

Tissue-resident memory T cells (Trm) belong from peripheral Tem that have originally migrated and stabilized into a specific tissue; in the CNS, they hereby provide immune surveillance in non-pathological conditions. Trm can be altered in disease and have been questioned for their potential role in MS pathogenesis [197]. Trm are normally sustained by factors such as TGFβ, IL-33 and IL-15 [198]. Most of CNS Trm are CD8 + , express tissue-homing receptors i.e. CCR5 and CXCR6, and may produce CKs and granzyme upon stimulation, as defense from harmful agents such as viruses [199]. Kaufmann et al. suggested that Trm may be able to react against CNS-self antigens triggering an abnormal immune response, in concomitance with individual genetic and environmental susceptibility factors. This would support the hypothesis that the primary self-response that eventually leads to an autoimmune disease may happen at central level, within the CNS, rather than consequent to immune cell trafficking from the periphery. However, this issue is still matter of debate.

To sum up, CKs are physiologically produced by parenchymal glial cells [13, 19, 20] to modulate neurons and synapses functions, or by basal T-cell subsets circulating within the CSF to guarantee immune surveillance [47, 49, 50]. Neuroinflammation alters this balance occurring with immune cells crossing from the periphery through the impaired BBB and local activation of glial cells that produce detrimental amounts of CKs [46, 55–57]. The study of MS, a neuroinflammatory, demyelinating disease with a main role of T cells, and its murine model EAE, greatly contributed to the understanding of CKs role(s) in the affected CNS, also paving the way to explore novel potential therapeutical targets. To date, we know that abnormal production of CKs in MS patients impairs glutamatergic and GABAergic synapses with a clear impact in cognitive decline [101, 102], as disbalance in several CKs, i.e. TNF-α, IL-1β, and GM-CSF have been found in both MS and EAE model [107, 108, 118, 119]. On the other hand, the substantial infiltration of immune cells in the CNS of MS patients increases the local levels of pro-inflammatory CKs and recall chemokines fuelling further cell infiltration, demyelination, and overall chronic damage [128, 129]. Some CKs have been considered as potential targets in MS treatments, such as GM-CSF [158] and IL-10 [188, 189]. Unfortunately, not all trials reported promising results [151]. Other CKs were never introduced in any trial and may be in the future, especially in studies involving synaptic modulation, particularly important in MS forms with a prominent cognitive decline. Overall, insights on the role of CKs in CNS disease pathogenesis and on the possibility to use them as therapeutic targets is worthwhile of future investigations.

## Data Availability

Data sharing is not applicable to this article as no datasets were generated or analysed during the current study.

## Abbreviations

AMPAR: AMPA receptors
APC: Antigen presenting cells
BBB: Blood–brain barrier
CCR: C–C chemokine receptor
CCL: C–C chemokine ligand (CCL)
CFA: Complete Freund’s adjuvant
CNS: Central nervous system
CKs: Cytokines
CSF: Cerebrospinal fluid
CXCL: C-X-C motif chemokine ligand
CXCR: C-X-C motif chemokine receptor
EAE: Experimental autoimmune encephalomyelitis
EBV: Epstein-Barr virus
ECs: Endothelial cells
ECM: Extracellular matrix
GA: Glatiramer acetate
GM-CSF: Granulocyte–macrophage colony-stimulating factor
GPCRs: G-protein-coupled receptors
HLA: Human leukocyte antigen
ICAM-1: Intracellular adhesion molecule 1
IFN-γ: Interferon-γ
Ig: Immunoglobulin
IL: Interleukin
IL-1R: IL-1 receptor
LTP: Long term potentiation
LTD: Long term depression
LFA-1: Lymphocyte function-associated-1
MAPK: Mitogen activated protein kinase
MMPs: Metalloproteinases
MOG: Myelin oligodendrocyte glycoprotein
MS: Multiple sclerosis
NF-κB: Nuclear factor kappa-light-chain-enhancer of activated B cells
NMDAR: NMDA receptors
NVU: Neurovascular unit
OCBs: Oligoclonal bands
PD-L1: Programmed death-ligand 1
PLP: Myelin proteolipid protein
PSGL-1: P-selectin glycoprotein ligand 1
RRMS: Relapsing–remitting multiple sclerosis
SERT: Serotonin transporter
SNPs: Single-nucleotide polymorphisms
SPMS: Secondary-progressive multiple sclerosis
Tcm: Central memory T cells
Tem: Effector memory T cells
Th: T helper cells
TNF-α: Tumor necrosis factor-α
TNF-R1: TNF-receptor 1
TGFβ: Transforming growth factor β
Trm: Resident memory T cells
TRPV1: Transient receptor potential vanilloid 1
VLA-4: Very late antigen-4
